# Supplementary material for: ascend: R package for analysis of single-cell RNA-seq data
Source: Gigascience. 2019 Aug 24;8(8):giz087. doi: 10.1093/gigascience/giz087 (PMC6735844; doi:10.1093/gigascience/giz087)
Supplement: giz087_GIGA-D-19-00140_Original_Submission [file giz087_giga-d-19-00140_original_submission.pdf]

GigaScience

ascend: R package for analysis of single cell RNA-seq data

--Manuscript Draft--

**Manuscript Number:**	GIGA-D-19-00140
**Full Title:**	ascend: R package for analysis of single cell RNA-seq data
**Article Type:**	Technical Note
**Funding Information:**	
**Abstract:**	Background Recent developments in single cell RNA sequencing (scRNA-seq) platforms have vastly increased the number of cells typically assayed in an experiment, as well as accessibility of the technology itself. Few analysis packages exist that are at once robust, computationally fast, and allow flexible integration with other bioinformatics tools and methods. Findings ascend is an R package comprised of tools designed to simplify and streamline the preliminary analysis of scRNA-seq data, while addressing the statistical challenges of scRNA-seq analysis, while enabling flexible integration with genomics packages and native R functions, including fast parallel computation and efficient memory management. The package incorporates both novel and established methods to provide a framework to perform cell and gene filtering, quality control, normalization, dimension reduction, clustering, differential expression, and a wide-range of visualization functions. Conclusions ascend is designed to work with scRNA-seq data generated by any high-throughput platform, and includes functions to convert data objects between software packages. ascend workflow is simple and interactive, suitable for implementation by a broad range of users, including those with little programming experience.
**Corresponding Author:**	Joseph Powell<br><br>AUSTRALIA
**Corresponding Author Secondary Information:**	
**Corresponding Author's Institution:**	
**Corresponding Author's Secondary Institution:**	
**First Author:**	Joseph Powell
**First Author Secondary Information:**	
**Order of Authors:**	Joseph Powell
**Order of Authors Secondary Information:**	
**Additional Information:**	
**Question**	**Response**
Are you submitting this manuscript to a special series or article collection?	No
**Experimental design and statistics**<br><br>Full details of the experimental design and statistical methods used should be given in the Methods section, as detailed in our Minimum Standards Reporting Checklist. Information essential to interpreting the data presented should be made available in the figure legends.	No

Powered by Editorial Manager® and ProduXion Manager® from Aries Systems Corporation

Have you included all the information requested in your manuscript?	
If not, please give reasons for any omissions below.<br><br>as follow-up to "**Experimental design and statistics**<br><br>Full details of the experimental design and statistical methods used should be given in the Methods section, as detailed in our Minimum Standards Reporting Checklist. Information essential to interpreting the data presented should be made available in the figure legends.<br><br>Have you included all the information requested in your manuscript?<br><br>"	This manuscript is describing a software package
**Resources**<br><br>A description of all resources used, including antibodies, cell lines, animals and software tools, with enough information to allow them to be uniquely identified, should be included in the Methods section. Authors are strongly encouraged to cite Research Resource Identifiers (RRIDs) for antibodies, model organisms and tools, where possible.<br><br>Have you included the information requested as detailed in our Minimum Standards Reporting Checklist?	No
If not, please give reasons for any omissions below.<br><br>as follow-up to "**Resources**	no resources are used

A description of all resources used, including antibodies, cell lines, animals and software tools, with enough information to allow them to be uniquely identified, should be included in the Methods section. Authors are strongly encouraged to cite Research Resource Identifiers (RRIDs) for antibodies, model organisms and tools, where possible.<br><br>Have you included the information requested as detailed in our Minimum Standards Reporting Checklist?<br><br>"	
**Availability of data and materials**<br><br>All datasets and code on which the conclusions of the paper rely must be either included in your submission or deposited in publicly available repositories (where available and ethically appropriate), referencing such data using a unique identifier in the references and in the "Availability of Data and Materials" section of your manuscript.<br><br>Have you have met the above requirement as detailed in our Minimum Standards Reporting Checklist?	Yes

Powered by Editorial Manager® and ProduXion Manager® from Aries Systems Corporation

Manuscript

Click here to download Manuscript ascend__single_cell_RNA_seq_R_package_revision.pdf

GigaScience, 2018, 1–6

doi: xx.xxxx/xxxx
Manuscript in Preparation
Technical Note

TECHNICAL NOTE

ascend: R package for analysis of single cell RNA-seq data

Anne Senabouth[1], Samuel W. Lukowski[2,3], Jose Alquicira Hernandez[1,2], Stacey B. Andersen[2], Xin Mei[2,4], Quan H. Nguyen[2†] and Joseph E. Powell[1,2,5*†]

[1]Garvan Institute of Medical Research, Sydney, Australia and [2]Institute of Molecular Bioscience, University of Queensland, Brisbane, Australia and [3]The University of Queensland Diamantina Institute, Translational Research Institute, Brisbane, Australia and [4]South China Botanical Garden, Chinese Academy of Sciences, Guangzhou, China and [5]Queensland Brain Institute, University of Queensland, Brisbane, Australia

[*]Corresponding author: j.powell@garvan.org.au
[†]Joint senior authors

Abstract

Background Recent developments in single cell RNA sequencing (scRNA-seq) platforms have vastly increased the number of cells typically assayed in an experiment, as well as accessibility of the technology itself. Few analysis packages exist that are at once robust, computationally fast, and allow flexible integration with other bioinformatics tools and methods. **Findings** *ascend* is an R package comprised of tools designed to simplify and streamline the preliminary analysis of scRNA-seq data, while addressing the statistical challenges of scRNA-seq analysis, while enabling flexible integration with genomics packages and native R functions, including fast parallel computation and efficient memory management. The package incorporates both novel and established methods to provide a framework to perform cell and gene filtering, quality control, normalization, dimension reduction, clustering, differential expression, and a wide-range of visualization functions. **Conclusions** *ascend* is designed to work with scRNA-seq data generated by any high-throughput platform, and includes functions to convert data objects between software packages. *ascend* workflow is simple and interactive, suitable for implementation by a broad range of users, including those with little programming experience.

Key words: single cell; scRNA-seq; filtering; clustering; normalization; differential expression; data visualization; R package

Findings

Background

Single cell RNA sequencing (scRNA-seq) has revolutionized the way we understand the transcriptional programs of cells. Recent advances in barcoding molecular biology techniques, coupled with microfluidics have yielded platforms such as 10x Genomics Chromium [1] and Drop-seq [2], which are capable of capturing the transcriptomes of tens of thousands of single cells simultaneously. The increased capacity of scRNA-seq has been advantageous as larger sample sizes provide greater statistical power, and correspondingly higher resolution to determine differences in cellular features. A consequence has been the increase in the complexity of scRNA-seq data, creating new challenges for data management, statistical methods, data visualization, and computing strategies. A number of scRNA-seq specific methods and toolkits have been developed to address these challenges ([3], [4] and [5]), but both the functionality, and specific methods implemented vary. It is becoming appar-

Compiled on: April 10, 2019.
Draft manuscript prepared by the author.

Key Points

- *ascend* is a fast and easy-to-use software for thorough and interactive analysis of scRNA-seq data.
- *ascend*'s streamlined workflow includes filtering, normalization, dimension reduction, clustering, differential expression and visualization.
- *ascend* optimizes parallelization and algorithms for improving speed of each analysis step. e.g. differential expression analysis
- *ascend* implements Clustering by Optimal REsolution (CORE) for unsupervised, robust hierarchical clustering.

ent that for a given scRNA-seq dataset, the specific analysis steps need to be carefully considered in light of the underlying biology. For single cell analysis packages, flexibility in both the choice of methods implemented and arguments passed to functions is therefore important.

Here we present *ascend*, an R package designed to create a simple and streamlined workflow for the analysis of scRNA-seq experiments. *ascend* is designed to handle data generated from any single cell library preparation platform; this can include data from single and paired-end reads, and optionally, with unique molecular identifiers (UMIs). *ascend* imports scRNA-seq data following the generation of an expression matrix consisting of transcript counts from each cell, and performs user-friendly quality control, filtering, normalization, dimension reduction, clustering, differential expression and visualization. It includes functions to leverage multiple CPUs, allowing most analyses to be performed on a standard desktop or laptop.

Data object

The foundation of the *ascend* R package is the **E**xpression and **M**etadata **Set**, a data container class that inherits from the SingleCellExperiment superclass [6]. The SingleCellExperiment class, from the Bioconductor R package of the same name, was introduced as a container class specifically for single cell genomics data. It is structured in the context of the gene-cell expression matrix and contains slots that can hold data that may be used in scRNA-seq analysis - specifically spike-in information, normalization factors, transformations of the original count data, metadata and data related to cells and genes.

The EMSet deviates from the SingleCellExperiment in which it is a dynamic element. The object is always accompanied by a set of quality control metrics that is reflective of the data that is currently stored in the counts slot of the object. These values are automatically recalculated by the package whenever changes are made to the count matrix, which occurs during batch normalisation and filtering. Another feature of the EMSet is the logging of operations, ensuring analysis is performed in the correct order and allowing users to review changes. As metadata can play a key role in functions such as plotting and differential expression analysis, we have separated cell-related and gene-related metadata from calculated values by storing them in dedicated slots introduced by the EMSet. Additional slots have also been introduced to store objects related to clustering and differential expression analysis.

The EMSet retains the convenient row and column subsetting operations of the SingleCellExperiment and introduces methods to manipulate the object based on conditions defined in the cell metadata slot. To ensure compatibility with other software packages that also use the SingleCellExperiment class, a conversion function is supplied to preserve data stored in EMSet-specific slots. This data can then be retrieved when converting back to an EMSet.

Batch normalization

Typically, samples comprising of libraries of thousands of cells are often processed in separate batches and the resulting data require aggregation before analysis. This can introduce systematic biases due to technical variation in each batch. To address this, *ascend* provides simple and fast methods to normalize between batches, (*normaliseBatches*). To perform fast batch-to-batch normalisation, we calculate a scaling factor for each batch and multiply the expression values to a batch-specific constant. The batch scale factor is the ratio of the median sequencing reads among all batches to the total reads of a batch. This scaling approach is more robust to scaling the higher-read depth batches to the one with the lowest read depth. Optionally, after batch-to-batch normalisation, we also perform cell-to-cell normalisation within a batch, using the same scaling method, but each cell now is equivalent to a batch. A scaling factor for a cell is the ratio of the median total reads among all cells to the total reads of the cell. This cell-to-cell scaling approach is more robust to scaling the total reads of the cell to a fixed constant, such as in the approach to scaling all cells to the library depth of 10,000 reads per cell as applied in the Seurat workflow. [3]

Filtering and quality control

Quality control (QC) is an important step of scRNA-seq data analysis, as it can be used to reduce poor quality data that may mask biologically significant variation [7]. Sources of technical noise include low quality cells that are generally defined as empty droplets, droplets with multiple cells, and dead or dying cells [8]. The quality of cells is described by a series of metrics, such as total number of reads, number of genes expressed by a cell, mean gene expression of a gene or a cell and proportion of a gene's expression to total expression. Low quality cells are identified as outliers in terms of library size and gene expression or with expression dominated by controls that are usually defined as mitochondrial and ribosomal genes. As the EMSet automatically recalculates QC metrics when changes are made to the count matrix, the quality of the dataset can be monitored in real-time with the aid of quality control plots (*plotGeneralQC*). Users can also review the EMSet log for a record of cells and genes removed by filtering methods. Since these QC steps should allow a user to filter cells or genes based on their own defined metrics, *ascend*'s QC functions allow arguments to be passed using additional metadata.

Cell-cell normalization

Cell-cell normalization is another crucial step to remove technical variation between individual cells. The normalization of scRNA-seq data is complicated by the zero-inflated count distributions of genes, that may be due to either biological or technical factors. *ascend* addresses this issue by adapting the

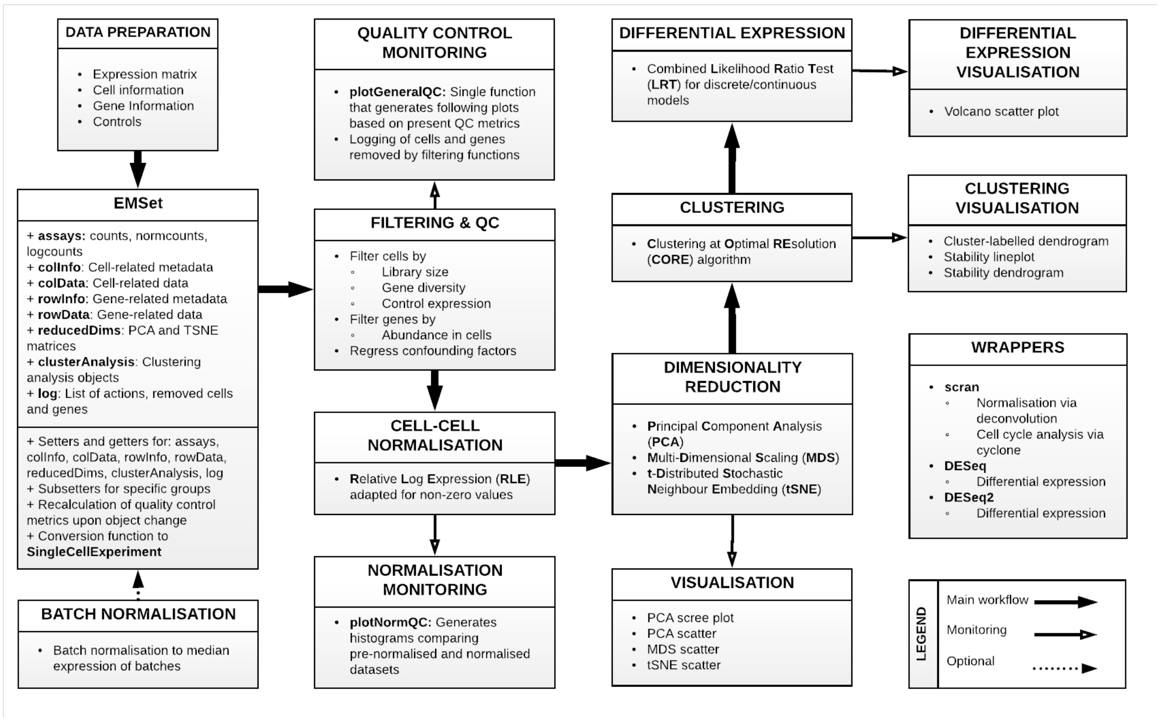

Figure 1. A summary of the typical analysis workflows and major function groups available in *ascend*.

normalization by Relative Log Expression (RLE) method[9] for zero-inflated data by estimating size factors from the geometric means based on true count values that are greater than zero. For each gene, a gene-specific geometric mean is estimated across all cells, not including cells with zero values. The cell-specific size factor is then calculated based on the expression of the gene in a cell relative to the geometric mean of that gene. The size factors for all genes in a cell are used for calculating the cell-specific size factor. We introduce the use of scran wrapper function as the default normalisation method, and recommend this method if the computation time and memory are not limiting factors. Alternatively, the RLE approach introduced here is the faster and more memory efficient option for cell-to-cell normalisation. Users can review the impact of normalization on the counts by generating a series of plots with the *plotNormQC* function, that compares pre-normalized and normalized library sizes and individual gene counts.

Reduction of high-dimensional space

Since scRNA-seq data is typically multiple orders of magnitude larger than bulk RNA-seq data (*n*-cells x *m*-genes), dimensionality reduction is vital. Moreover, the expression levels of many genes are likely to be correlated, therefore the problem of collinearity is common, while additional factors such as dropout rate, and high expression variation increase noise in the data [10]. *ascend* contains functions to perform principal component analysis (PCA) to reduce the dimensions of the normalized count data and preserve the data structure (i.e. explain the majority of the variance between cells). t-SNE (t-distributed Stochastic Neighbor Embedding) and Multi-Dimensional Scaling (MDS) are only used to visualize cells in a low-dimensional space, supplemented by information supplied by the user or generated by downstream analysis.

Clustering

Clustering cells into subpopulations or subtypes provides structure to the dataset by grouping transcriptionally similar cells. *ascend* implements our previously published CORE method [11], which identifies the most stable clustering identity. Firstly, a Euclidean distance matrix between cells is calculated from the first 20 principal components of the PCA-reduced normalized count matrix. An unsupervised dendrogram is then constructed by applying hierarchical clustering. Outlier cells identified by this initial round of clustering removed from the dataset, although their identifiers are retained in the EMSet logs. The dendrogram is then dynamically re-clustered by a top-down split and merging process over multiple iterations with changing tree-height thresholds. This approach merges smaller clusters into larger consensus clusters, and uses an adjusted Rand index to compare different clustering results to identify the most stable number of clusters. The method is fast and scalable, enabling the analysis of small clusters at high resolution, or larger clusters for more general classification with simpler downstream analysis.

Differential expression

In a heterogeneous dataset, such as scRNA-seq data, analyzing the differentially expressed (DE) genes between one cluster and the combined remaining clusters can reveal signature genes that can be used to assign identity to a population of cells, or to more clearly understand cell transition states. After decomposing the data into subpopulations, *ascend* provides a combined Likelihood Ratio Test (LRT) to compare these subpopulations by finding biological signatures that distinguish them, taking into account subpopulation-size imbalance and high drop-out rates. Introduced as a method for single-cell qPCR data[12], the combined LRT has been adapted in *ascend* such that it takes into account genes with zero variance. LRT is especially suitable for the cases where the number of cells in two clusters are very different. In these cases, most dispersion

estimation methods, such as those in DESeq donot result in a convergence. The imbalance issue becomes exaggerated for the cases of smaller clusters, where the high drop-out rates have a higher impact. LRT uses a combined distribution assumption consisting of both discrete (on/off) and continuous (low/high expression) components, which helps overcome the issues in dropout and small number of cells. The resulting implementation is fast, scalable and robust, even in situations where standard DE methods fail. Wrapper functions are also provided for DE analysis based on negative binomial tests from DESeq (Anders and Huber, 2010). We introduced several modifications that allow (i) more accurate estimation of fold change (adjusted fold change), and (ii) more efficient multiprocessing, using a divide and conquer approach, to handle large datasets and substantially reduce computational time.

Benchmarking

The CPU time of the ascend package was compared to two other toolkits developed for scRNA-seq analysis - *Seurat*[13] and *scater*[4]. Using a dataset that comprised of 1,272 retinal ganglion cells from the study by Daniszewski et al. [14], these packages were used to perform quality control, normalization, dimensionality reduction, clustering and differential expression using equivalent methods. As shown in Supplementary File 1, ascend's processing time is comparable to *Seurat*[13] and *scater*[4].

Conclusion

In summary, *ascend* is a user-friendly and computationally efficient package for analyzing scRNA-seq data from all experimental platforms. *ascend* implements quality control and filtering approaches that are highly customizable, a state-of-the-art unsupervised clustering method (SCORE), and optimizes speed for implementing established analysis techniques for normalization and differential gene expression. The *ascend* package and context-specific tutorials addressing a range of analytical scenarios are available at https://github.com/IMB-Computational-Genomics-Lab/ascend. We expect that *ascend* is especially useful for biologists who wish to explore their own datasets using expert domain knowledge and an easy-to use and complete toolkit.

Methods

Data

Here we present an application case study of *ascend* using scRNA-seq data from undifferentiated human induced Pluripotent Stem Cells (hiPSCs) generated as described by Nguyen and Lukowski *et al.* [11]. The raw 10x Chromium Single Cell 3' Gene Expression dataset consists of 20,448 cells that are divided into five samples. Raw (FASTQ or aggregated count matrix) and processed data can be downloaded from ArrayExpress (accession number: E-MTAB-6687).

Preprocessing of scRNA-seq dataset

The raw expression data from each sample were combined into a single dataset using Chromium's Cell Ranger 1.2.0 *aggr* function. This function performs two tasks - batch normalization and transcript count aggregation. Cell Ranger first normalizes the sequencing depth between the five samples by subsampling reads for each sample until their median depth equals the sample with the shallowest read depth. Once normalized, the transcript counts from each sample are combined into a single matrix. The rows of this matrix were labeled with ENSEMBL gene identifiers; to simplify analysis, these were replaced with corresponding gene names that were stored in the Cell Ranger outputs.

"ascend" analysis

ascend was run in RStudio (R Version 3.5.0), and analysis of data from quality control to differential expression (LRT) took 95 minutes on a MacBook Pro laptop with a dual-core Intel Core i5 2.7GHz and 8GB of RAM. To minimize memory use, the raw expression matrix was converted into a sparse matrix using the Matrix R Package [15]. The sparse matrix and accompanying cell-related metadata were loaded into an *EMSet*.

```
# Build an EMSet
EMSet <- newEMSet(assays = list(counts = expression_matrix),
colInfo = colInfo,
rowInfo = rowInfo,
controls = controls)
```

The quality of the expression data was assessed with the aid of quality control figures generated by the *plotGeneralQC* function from *ascend* package.

```
raw_qc_plots <- plotGeneralQC(EMSet)
```

The data then underwent quality control. First, cells were filtered based on library size, number of detected genes and reads mapped to mitochondrial and ribosomal genes using the default threshold of 3 x MAD range. Next, cells were removed if 20% of reads were mapped to mitochondrial transcripts and 50% of reads were mapped to ribosomal transcripts. Finally, genes were removed if they were expressed in less than 0.1% of the cell population.

```
# Remove cells that are outliers
EMSet <- filterByOutliers(EMSet,
cell.threshold = 3,
control.threshold = 3)
# Remove cells where mitochondrial-related
# transcripts account for at least 20% of reads
EMSet <- filterByControl(EMSet,
control = "Mt",
pct.threshold = 20)
# Remove cells where ribosomal-related
# transcripts account for at least 50% of reads
EMSet <- filterByControl(EMSet,
control = "Rb",
pct.threshold = 50)
# Remove genes that are expressed in less than
# 0.1% of the cell population
EMSet <- filterLowAbundanceGenes(EMSet, pct.threshold = 0.1)
```

QC removed 1,681 cells and 15,849 genes, leaving 18,767 cells and 16,889 genes for further analysis. The UMI counts for the remaining cells and genes were normalized with the *normaliseByRLE* function. The effectiveness of the normalization method was assessed with the aid of figures generated by the *plotNormQC* function. Mitochondrial and ribosomal gene transcripts were removed from the data before proceeding with further analysis.

```
# Normalize dataset using RLE
EMSet <- normaliseByRLE(EMSet)

# Plot normalisation quality control plots
```

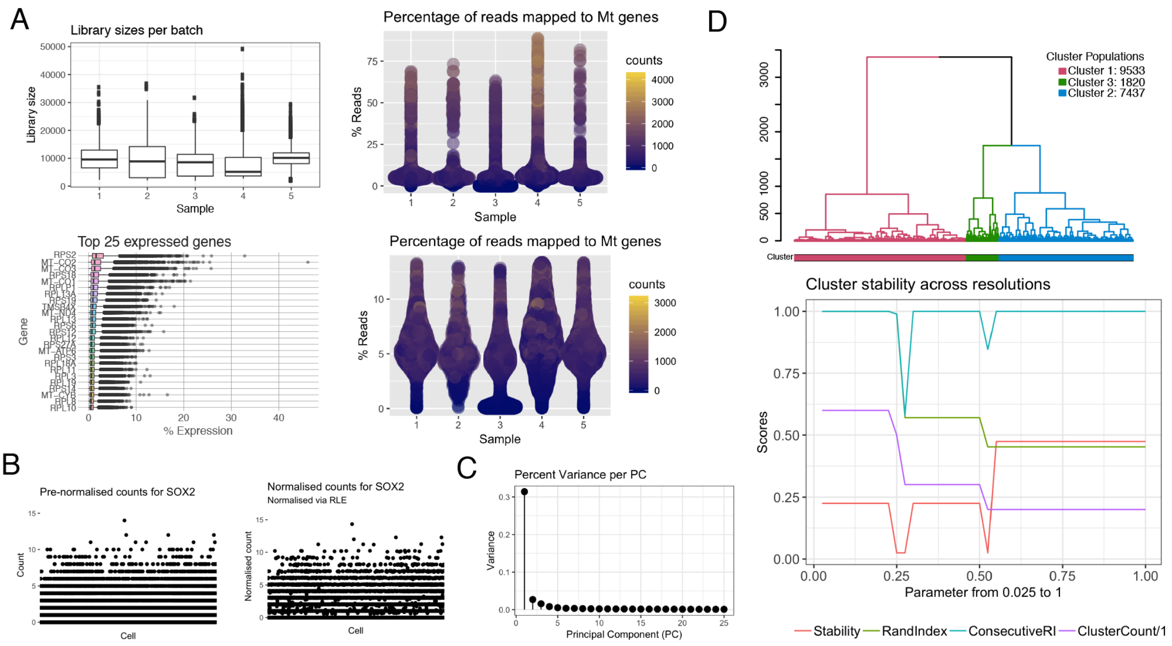

Figure 2. Graphics generated by *ascend* during different stages of analysis. A: Quality control plots include a boxplot representing distribution of library sizes across each batch, a boxplot representing the expression of the top 25 most abundant transcripts and violin plots representing proportion of mitochondrial-related transcripts to total expression per sample. B: Normalization quality control plot represents the expression of the SOX2 gene before and after RLE normalization. C: Scree plot related to Principal Component dimensionality reduction. D: Clustering plots include a cluster-labeled dendrogram and a line plot depicting the relationships between cluster numbers and stability.

```
norm_qc <- plotNormQC(EMSet)

# Remove controls from dataset
EMSet <- excludeControl(EMSet, control = c("Mt", "Rb"))
```

To reduce the dimensions of the data, the normalized UMI count matrix was reduced using the *ascend* function *runPCA*. This function is a wrapper for R's *prcomp* function.

```
# Reduce dataset with PCA
EMSet <- runPCA(EMSet, ngenes = 1500, scaling = TRUE)
```

The scree plot generated by *ascend*'s *plotPCAVariance* function revealed the first 5 principal components (PCs) explained 38.44% of the variance in this data. These 5 PCs were passed to the CORE algorithm function to build a cell distance matrix, and subsequently a dendrogram that was used to identify clusters.

```
EMSet <- runCORE(EMSet,
conservative = FALSE,
nres = 40,
dims = 10,
remove.outlier = TRUE)
```

Using the default arguments, the CORE method generated clustering results for 40 different resolutions, and based on the Rand index, the function identified three clusters of cells that represent the most stable result. Clusters 1, 2 and 3 comprised of 9073, 7240 and 2477 cells respectively.

To characterize the biological properties of the three clusters, differential expression was performed using *ascend*'s *runDiffExpression* function. The expression of each cluster was compared to the expression of the other clusters.

```
# Comparison of cluster 1 vs other clusters
cluster1_vs_all <- runDiffExpression(EMSet,
group = "cluster",
    condition.a = 1,
    condition.b = c(2, 3, 4))

# Comparison of cluster 2 vs other clusters
cluster2_vs_all <- runDiffExpression(EMSet,
group = "cluster",
    condition.a = 2,
    condition.b = c(1, 3, 4))

# Comparison of cluster 3 vs other clusters
cluster3_vs_all <- runDiffExpression(EMSet,
group = "cluster",
    condition.a = 3,
    condition.b = c(1, 2, 4))

# Comparison of cluster 3 vs other clusters
cluster4_vs_all <- runDiffExpression(EMSet,
group = "cluster",
    condition.a = 4,
    condition.b = c(1, 2, 3))
```

Using a Bonferroni-corrected P-value threshold ($P < 3.1 \times 10^{-7}$) and an absolute $\log_2$ fold change greater than 2, differential expression analysis revealed clusters 1, 2, and 3 respectively had 269, 22, and 198 differentially expressed genes.

Availability of source code and requirements

- Project name: *ascend*
- Project home page: https://github.com/IMB-Computational-Genomics-Lab/ascend
- Operating system(s): Platform independent
- Programming language: R
- Other requirements: R 3.5, Bioconductor 3.7
- License: GPL 3.0

Availability of supporting data and materials

The data supporting the results of this article are available in ArrayExpress at https://www.ebi.ac.uk/arrayexpress/experiments/E-MTAB-6687/.

Declarations

List of abbreviations

scRNA-seq: single cell RNA-sequencing; RNA-seq: RNA-sequencing; CORE: clustering at optimal resolution; PCA: principal component analysis; t-SNE: t-distributed Stochastic Neighbor Embedding; MDS: multi-dimensional scaling; RLE: relative log expression; UMI: unique molecular identifier.

Ethical Approval

Not applicable.

Consent for publication

Not applicable.

Competing Interests

The author(s) declare that they have no competing interests.

Funding

This work was supported by the National Health and Medical Research Council grants 1107599 and 1083405.

Author's Contributions

AS wrote the software; all authors contributed to software development; AS, SWL, QHN and JEP wrote the manuscript. QHN and JEP oversaw the project.

References

1. Zheng GXY, Terry JM, Belgrader P, Ryvkin P, Bent ZW, Ziraldo SB, et al. Massively parallel digital transcriptional profiling of single cells. Nature Communications 2017;8(206):667–3170.
2. Macosko EZ, Basu A, Satija R, Nemesh J, Shekhar K, Goldman M, et al. Highly parallel genome-wide expression profiling of individual cells using nanoliter droplets. Cell 2015;161(5):1202–1214.
3. Butler A, Hoffman P, Smibert P, Papalexi E, Satija R. Integrating single-cell transcriptomic data across different conditions, technologies, and species analysis. Nature Biotechnology 2018;36(5).
4. McCarthy DJ, Campbell KR, Lun ATL, Wills QF. Scater: Pre-processing, quality control, normalization and visualization of single-cell RNA-seq data in R. Bioinformatics 2017;33(8):1179–1186.
5. Lun ATL, Bach K, Marioni JC. Pooling across cells to normalize single-cell RNA sequencing data with many zero counts. Genome Biology 2016;17.
6. Lun A, Risso D. SingleCellExperiment: S4 Classes for Single Cell Data; 2018, r package version 1.3.6.
7. Bacher R, Kendziorski C, Auer P, Doerge R, Robles J, Qureshi S, et al. Design and computational analysis of single-cell RNA-sequencing experiments. Genome Biology 2016;17(1):63.
8. Ilicic T, Kim JK, Kolodziejczyk AA, Bagger FO, McCarthy DJ, Marioni JC, et al. Classification of low quality cells from single-cell RNA-seq data. Genome Biology 2016;17(1).
9. Anders S, Huber W. Differential expression analysis for sequence count data. Genome Biology 2010;11(10):R106.
10. Hicks SC, Townes FW, Teng M, Irizarry RA. Missing data and technical variability in single-cell RNA-sequencing experiments. Biostatistics 2017 Nov;.
11. Nguyen Q, Lukowski S, Chiu H, Senabouth A, Bruxner T, Christ A, et al. Single-cell RNA-seq of human induced pluripotent stem cells reveals cellular heterogeneity and cell state transitions between subpopulations. Genome research 2018 jul;28(7):gr.223925.117.
12. McDavid A, Finak G, Chattopadyay PK, Dominguez M, Lamoreaux L, Ma SS, et al. Data exploration, quality control and testing in single-cell qPCR-based gene expression experiments. Bioinformatics 2013 feb;29(4):461–467.
13. Satija R, Farrell JA, Gennert D, Schier AF, Regev A. Spatial reconstruction of single-cell gene expression data. Nature Biotechnology 2015;33(5):495–502. https://www.nature.com/articles/nbt.3192.pdf.
14. Daniszewski M, Senabouth A, Nguyen Q, Crombie DE, Lukowski SW, Kulkarni T, et al. Single Cell RNA Sequencing of stem cell-derived retinal ganglion cells. bioRxiv 2017 jan;http://biorxiv.org/content/early/2017/09/22/191395.abstract.
15. Bates D, Maechler M. Matrix: Sparse and Dense Matrix Classes and Methods; 2018, https://CRAN.R-project.org/package=Matrix, r package version 1.2-14.

Click here to access/download
Supplementary Material
Supporting_material_File1.xlsx

Cover Letter
Click here to download Personal Cover CoverLetter_ascend_revision.pdf

384 Victoria Street
Darlinghurst NSW 2010
Sydney, Australia

T +61 2 9295 8100
F +61 2 9295 8101
www.garvan.org.au

GARVAN INSTITUTE OF MEDICAL RESEARCH

Regarding GIGA-D-18-00359

Dear Dr Hans Zauner,

On behalf of my co-authors, we delighted to submit a revised version of our manuscript entitled "***ascend: R package for analysis of single cell RNA-seq data***" for consideration as a technical note in *GigaScience*.

Our manuscript was previously reviewed by two referees, who raised a number of helpful comments and questions. We have now addressed all of these points, and have included a point-by-point reply in this re-submission. In particular, the referees raised concerns about comparisons of both functions and computational performance to other single cell analysis software packages, and choice of certain statistical tools at key analysis steps. We have now benchmarked performance against both the *scater* and *seurat* packages, included new functions, and highlighted the inclusion of function to run new methods that have been developed by our group. Specifically, the SCORE clustering method (Nguyen et al. Genome Research 2018), and lineage development method scGPS (Friedman et al. Cell Stem Cell 2018; Nguyen et al. biorxiv: 229336).

ascend is a comprehensive R package designed to create a simple and streamlined workflow for the analysis of scRNA-seq experiments. *ascend* can read in and handle data generated from any single cell library preparation platform; which can include data from either single and pair-end reads, and including or not including unique molecular identifiers (UMIs). *ascend* imports scRNA-seq data following bioinformatics processing, performs user-friendly quality control, filtering, normalization, dimension reduction, clustering, differential expression and visualization. It includes functions to leverage multiple CPUs, allowing most analyses to be performed on a standard desktop or laptop. The package has class-specific convenience functions allowing object components to be manipulated in place or extracted with ease and standard R functions can be used to modify metadata. In addition, to promote cross-package compatibility, a convenience method is available to convert the ascend data objects to/from another common data classes, such as those used in *scater*, *scran,* and *seurat*.

The *ascend* package is already used widely, including for analysis in publications under review and on *bioRxiv*. The package has undergone Bioconductor review, and is available to freely download from https://github.com/IMB-Computational-Genomics-Lab/ascend. We have supplied a comprehensive user guide and made available an example dataset as a companion to the vignette.

Thank you for your consideration.

Yours sincerely,

Associate Professor Joseph Powell

Head | Garvan-Weizmann Centre for Cellular Genomics

Reply to reviewer comments

Click here to download Personal Cover Reply_to_Referee_Comments.pdf

Referee #1:

1. I've read the manuscript about the ascend R package. It is very well written and everything is explained. However, my major concern is that in terms of applications it is not really different from the already published scater package. The only real difference I can see is that the log is also stored in the data object. Therefore, since the developers of scater tried to optimise it very much by using C code where possible, it would be important to compare the running times of ascend and scater on some of the common functions and to show in which cases it is more advantageous to use either ascend or scater.

We would like to thank the referee for taking time to review our manuscript and providing helpful comments and suggestions to improve our software package and manuscript. We appreciate that there is a degree of overlap in some of the functions between *ascend* and other packages such as *scater* and *Seurat*. However, we would like to make it clear that *ascend* implements a new statistical method developed and published by our team - specifically the SCORE clustering method (Nguyen et al. Genome Research 2018), and lineage development method scGPS (Friedman et al. Cell Stem Cell; Nguyen et al. biorxiv: 229336). In addition, we have included a simplified, streamlined approach to quality control, filtering and normalisation.

The EMSet object deviates from the SingleCellExperiment in which it is a dynamic element; the object is always accompanied by a set of quality control metrics that is reflective of the data that is currently stored in the counts slot of the object. These values are automatically recalculated by the package whenever changes are made to the count matrix. Also, cell-related and gene-related metadata can be separated from calculated values by storing them in dedicated slots introduced by the EMSet. We acknowledge the SingleCellExperiment has the metadata slot for such information, but we feel this information should be structured in a manner that is compatible, and remain synchronised with the data slots. Furthermore, we do not intend *ascend* to be a static package, and will add functionality as new methods are either developed by our team, or other approaches that become generalisable.

However, we do appreciate the points regarding computational resource benchmarking against similar functions in other packages and have performed comparative benchmarking of the following common functions - data loading, filtering and dimensionality reduction via Principal Component Analysis (PCA), clustering and differential expression analysis between *ascend, scater* and *Seurat* packages. Benchmarking was performed on equivalent functions where possible. Of note, *Scater* does not have equivalent functions for DE analysis and clustering. Our results are included in the 'Benchmarking' section and Supplementary File 1. Using a dataset comprised of 1,272 cells and 33,020 genes - we demonstrated *ascend*'s computational performance is comparable to that of *Seurat* and *scater.* We devised new approaches to achieve fast speed and light memory requirement for several key analysis. For example, we introduced a "split and conquer" approach to perform differential expression (DE) analysis, which is among the most popular but computationally intensive tasks. The benchmarking shows that *ascend* is faster the *Seurat* in DE analysis. Moreover, optimisation of functions in *ascend* stems from the use of the *Matrix* R package that uses a C backend for operations on sparse matrices, and parallelization via BiocParallel, and fast PCA using *irlba* bidiagonalization algorithm. Collectively, these steps reduce the required CPU hours and maximum memory allocation needed, making analysis of larger datasets more feasible.

We would also like to add that since our original submission we have further developed the code base for ascend and the package is currently undergoing bioconductor review.

2. In addition, I don't fully agree with the argument in the Introduction that all of the analysis tools should be part of the same package. I think that common data structures, such as SingleCellExperiment class give the developers freedom to develop various algorithms without worrying too much about compatibility and therefore there is no real need of having all methods used for the analysis in just one package.

We appreciate the point that the use of common data structures such as SingleCellExperiment help support developers use of different tools or packages when conducting analyses, and have amended the text to reflect this (Introduction). The EMSet is an extension of the SingleCellExperiment, and we have included conversion functions for other scRNA-seq R packages - specifically *Seurat, DESeq2* and *SCONE.* In our original manuscript we did not directly present an argument that all analysis tools should be part of the same package. However, we do believe that in a number of instances where having a single package is beneficial. This is particularly the case for less experienced computational analysts, or those new to the field. Such tools are especially useful for biologists who wish to explore their own data using their expert domain knowledge and an easy-to use tool kit. An example of this is the need to do data normalisation before clustering and differential analysis or trajectory analysis. We have added text in the Introduction to clarify the use cases for our package. We also feel that class objects such as SingleCellExperiment do not always facilitate ease of analyses for a given dataset between packages. For example, in developing the ascend package, the SingleCellExperiment class required expansion because we found one of our algorithms required access to outputs that was unable to be stored in the SingleCellExperiment object. Another example is the data encoding the tree structure from a clustering algorithm, where graphs are not stored in a SCE object, limiting visualization and interactive functionality.

Reviewer #2

1. The manuscript describes an R package (ascend) for analyzing single-cell RNA-seq data. The package works like a pipeline, containing methods to perform each step of the analysis (QC, normalization, clustering, etc.). Overall, the package works nicely and the plotting functions are useful. However, in a number of places the authors appear to have ignored previous work, which risks perpetuating inefficient analysis methods. Given that this package will work like a pipeline for users not aware of every single-cell methodology paper, it's important that the authors either provide justification or use the more relevant methods mentioned below.

We would like to thank the referee for providing useful comments on feedback on our work. We have addressed all comments and included a response to each point below and edited the manuscript, software package and user guide accordingly. We would also like to add that since our original submission we have further developed the code base for ascend and the package is currently undergoing bioconductor review.

2. The manuscript states that "Counts between batches are normalized by scaling to the median expression of all batches, while counts within a batch are normalized by scaling counts to the total expression of the batch." The meaning of this statement is unclear (especially the latter part). What if the median is zero? What is the difference between normalizing cells within a

batch (latter half of the above quote) and cell-cell normalization? Is this approach to batch correction sufficiently appropriate? Can the authors cite a relevant paper to back this up?

We apologize for the lack of clarity in our methods. We have now edited the manuscript in the 'cell-cell normalisation' and 'batch normalisation' sections to address these comments. For the normalisation step genes with a median or variance expression level of zero (i.e. not expressed in a dataset) would not be counted and thus excluded from influencing the normalisation of other genes. Of course, our user guide has recommended that these genes are removed prior to normalisation anyway. We have revised the manuscript which previously stated "Counts between batches are normalized by scaling to the median expression of all batches, while counts within a batch are normalized by scaling counts to the total expression of the batch." to "To perform fast batch-to-batch normalisation, we calculate a scaling factor for each batch and multiply the expression values to this batch-specific constant. A batch scale factor is the ratio of the median sequencing reads among all batches to the total reads of the batch. This scaling approach is more robust compared to a commonly used method that reduce the size of the higher-read depth batches to the one with the lowest read depth, resulting in the down-sampling of most of data. By using the median as a target read depth, both up-sampling and down-sampling are used among all the batches. We have also included an additional optional step after batch-to-batch normalisation, where the user can perform cell-to-cell normalisation within a batch, using the same scaling method, but each cell now is equivalent to a batch. A scaling factor for a cell is the ratio of the median total reads among all cells to the total reads of the cell. This cell-to-cell scaling approach is more robust to scaling the total reads of the cell to a fixed constant, such as in the approach to scaling all cells to the library depth of 10,000 reads per cell as applied in the Seurat workflow".

3. The authors proposed cell-cell normalization does not appear to be a good idea and should not be used. Specifically, the scran (Lun et al., 2016) paper states of this particular approach: "One might attempt to resolve the problem of stochastic zeroes by adding a pseudo-count prior to normalization. This would prevent biases due to unbalanced removal of zeroes between cells. However, direct addition of a pseudo-count squeezes all size factor estimates towards unity". The authors should also take a look at the 'scone' R package, which already thoroughly considers and checks a number of much more appropriate normalization methods. This package could contain a wrapper for specific functions from the scone package.'

We thank the referee for alerting us to the normalisation methods in the scone package. We agree that in many situations these methods may prove to be more appropriate ways of removing unwanted noise and normalising single cell data. We have now included a wrapper function in ascend to take advantage of the dependent scone package normalisation functions. This approach is mentioned in the user guide (available via the repository).

Regarding cell-cell normalisation, we believe that for the majority of datasets this approach is necessary and provides a robust way to correct for between cell differences in read depth. However, as with many analysis methods in single cell data, we do not believe this is the only approach, and should be chosen with some understanding of the biological properties of the data. We have cautioned as such in the user manual. However, we recognise that our original manuscript was unclear on the methods used and their justification. We have therefore revised the subsection cell-to-cell normalisation to clarify that pseudocount is not used. We emphasize the use of *scran* as the common normalisation method, when computation time is not an issue, and RLE is the alternative option for fast normalisation. However, we do not believe that RLE should replace *scran* in most instances. In RLE we also avoid adding 1 pseudo count. Instead,

0 counts are removed from calculating geometric means. In the scran normalisation procedure, adding pseudo-count is not included. We have processed over a hundred datasets and we observe no cases where the normalisation factors are shrunken towards unity. In a recent paper (Buttner et al. Nature Methods 16, 43–49 2019), the authors found *scran* outperforms other methods in removing batch effects. The *Scone* paper compares two top performing single cell normalisation method *scnorm* and *scran* with other non-single cell methods, suggesting equal performance, not to exclude *scran* as a bad option.

4. How is the authors extension of the combined LRT for sc-qPCR different from MAST? See:
https://www.ncbi.nlm.nih.gov/pmc/articles/PMC4676162/
https://bioconductor.org/packages/release/bioc/html/MAST.html
What is the justification of choosing to use a method for sc-qPCR data rather than one specifically for scRNA-seq data?If they are similar, then the additional citations need to be added.

The likelihood ratio test is especially suitable for the cases where the number of cells in two classes of a differential expression analysis are imbalanced (McDavid et al. Bioinformatics, 29, 461, 2013). In these cases, most dispersion estimation methods, such as those in DESeq do not result in convergence of the model. The imbalance issue becomes exaggerated for the cases where there is a very small number of cells in one class, where the drop-out rate has a higher impact. LRT uses a combined distribution assumption consisting of both discrete (on/off) and continuous (low/high expression) components, which helps overcome the issues in dropout and small number of cells. We have amended the text in 'differential expression' section to provide justification for this method.

5. The abstract states that the package implements both "novel and established methods". Which methods are novel? Given that no comparisons to other methods is done in the manuscript, the package should stick to established methods.

The ascend package included standard functions for things like cell and gene quality control, PCA, t-SNE and UMAP. However, it also includes the functions implementing new statistical methods developed and published by our team. In particular, our SCORE clustering method (Nguyen et al. Genome Research 2018) and our cell population trajectory method scGPS (Friedman et al. Cell Stem Cell; Nguyen et al. under revision biorxiv:229336). Furthermore, we do not intend *ascend* to be a static package, and will add functionality as new methods are either developed by our team, or other approaches that become generalisable.

6. The statement that "ascend is designed to handle data generated from any single cell library preparation platform; this can include data from single and paired-end reads, and optionally, with unique molecular identifiers (UMIs)" is a bit misleading. The statement almost implies the package does some aspect of the data pre-processing, unless any of the further steps in the package are relevant to differences between single- vs. paired-?

The *ascend* package is able to handle data generated from any platform, including single 3' assays through to full length paired end data. The point of ingest of data is after raw data has been demultiplex, reads assigned to a cell and aligned to a reference transcriptome. We have amended the text in 'Background' section to help make it clearer at which point in the data analysis journey that the ascend package functions start.

7. The manuscript states "Users can also review the EMSet log for a record of cells and genes removed by filtering methods." What criteria/function are used to filter genes?

Genes can be filtered based on their expression levels in either all cells, or subsets of cells. Functions are also included to both positively and negatively filter genes based on ontology or other functional classification. As an example of a niche application, a user could decide to only keep genes that were a known transcription factor or had a transcription factor binding site in a given encode cell type.

8. Some steps are missing from the vignette.

Example:

```
expression_matrix <- counts(datasets$RawSet)
# Pretend starting from scratch
em_set <- newEMSet(assays = list(counts = expression_matrix))
norm_matrix <- normcounts(em_set) # Error
plotGeneralQC(em_set) # Error
```

The two commands return an error because no normalization has been done and no QC. What is the function to normalize and QC filter in the package? What functions should be run in between? Or do col_info and row_info need to contain very specific sets of information? Neither was clear in the vignette itself.

We thank the reviewer for identifying this problem. This issue was caused by a bug in the output of a data object which has now been corrected.
