# Supplementary material for: ascend: R package for analysis of single-cell RNA-seq data
Source: Gigascience. 2019 Aug 24;8(8):giz087. doi: 10.1093/gigascience/giz087 (PMC6735844; doi:10.1093/gigascience/giz087)
Supplement: giz087_GIGA-D-19-00140_Revision_3 [file giz087_giga-d-19-00140_revision_3.pdf]

GigaScience

ascend: R package for analysis of single cell RNA-seq data

--Manuscript Draft--

**Manuscript Number:**	GIGA-D-19-00140R3
**Full Title:**	ascend: R package for analysis of single cell RNA-seq data
**Article Type:**	Technical Note
**Funding Information:**	
**Abstract:**	Background Recent developments in single cell RNA sequencing (scRNA-seq) platforms have vastly increased the number of cells typically assayed in an experiment, as well as accessibility of the technology itself. Few analysis packages exist that are at once robust, computationally fast, and allow flexible integration with other bioinformatics tools and methods. Findings ascend is an R package comprised of tools designed to simplify and streamline the preliminary analysis of scRNA-seq data, while addressing the statistical challenges of scRNA-seq analysis, while enabling flexible integration with genomics packages and native R functions, including fast parallel computation and efficient memory management. The package incorporates both novel and established methods to provide a framework to perform cell and gene filtering, quality control, normalization, dimension reduction, clustering, differential expression, and a wide-range of visualization functions. Conclusions ascend is designed to work with scRNA-seq data generated by any high-throughput platform, and includes functions to convert data objects between software packages. ascend workflow is simple and interactive, suitable for implementation by a broad range of users, including those with little programming experience.
**Corresponding Author:**	Joseph Powell<br><br>AUSTRALIA
**Corresponding Author Secondary Information:**	
**Corresponding Author's Institution:**	
**Corresponding Author's Secondary Institution:**	
**First Author:**	Joseph Powell
**First Author Secondary Information:**	
**Order of Authors:**	Joseph Powell
**Order of Authors Secondary Information:**	
**Response to Reviewers:**	Dear Hans,<br><br>the following action items have been completed.<br><br>1. the following has been added to the data availability section "An archival copy of the code and supporting data is also available via the GigaScience repository, GigaDB10.5524/100615"<br>2. A citation to the GigaDB doi to the bibliography has been added.<br>3. The ArrayExpress URL has been added to the bibliography<br><br>Many thanks,<br><br>// Joseph
**Additional Information:**	
**Question**	**Response**
Are you submitting this manuscript to a	No

Powered by Editorial Manager® and ProduXion Manager® from Aries Systems Corporation

special series or article collection?	
**Experimental design and statistics**<br><br>Full details of the experimental design and statistical methods used should be given in the Methods section, as detailed in our Minimum Standards Reporting Checklist. Information essential to interpreting the data presented should be made available in the figure legends.<br><br>Have you included all the information requested in your manuscript?	No
If not, please give reasons for any omissions below.<br><br>as follow-up to "**Experimental design and statistics**<br><br>Full details of the experimental design and statistical methods used should be given in the Methods section, as detailed in our Minimum Standards Reporting Checklist. Information essential to interpreting the data presented should be made available in the figure legends.<br><br>Have you included all the information requested in your manuscript?<br><br>"	This manuscript is describing a software package
**Resources**<br><br>A description of all resources used, including antibodies, cell lines, animals and software tools, with enough information to allow them to be uniquely identified, should be included in the Methods section. Authors are strongly encouraged to cite Research Resource Identifiers (RRIDs) for antibodies, model organisms and tools, where possible.	No

Have you included the information requested as detailed in our Minimum Standards Reporting Checklist?	
If not, please give reasons for any omissions below.<br><br>as follow-up to "**Resources**<br><br>A description of all resources used, including antibodies, cell lines, animals and software tools, with enough information to allow them to be uniquely identified, should be included in the Methods section. Authors are strongly encouraged to cite Research Resource Identifiers (RRIDs) for antibodies, model organisms and tools, where possible.<br><br>Have you included the information requested as detailed in our Minimum Standards Reporting Checklist?<br><br>"	no resources are used
**Availability of data and materials**<br><br>All datasets and code on which the conclusions of the paper rely must be either included in your submission or deposited in publicly available repositories (where available and ethically appropriate), referencing such data using a unique identifier in the references and in the "Availability of Data and Materials" section of your manuscript.<br><br>Have you have met the above requirement as detailed in our Minimum Standards Reporting Checklist?	Yes

Powered by Editorial Manager® and ProduXion Manager® from Aries Systems Corporation

Manuscript
Click here to view linked References
Click here to access/download;Manuscript;ascend_R_package_for_analysis

OXFORD (GIGA)^n SCIENCE

GigaScience, 2018, 1–6

doi: xx.xxxx/xxxx
Manuscript in Preparation
Technical Note

TECHNICAL NOTE

ascend: R package for analysis of single cell RNA-seq data

Anne Senabouth[1], Samuel W. Lukowski[2], Jose Alquicira Hernandez[1,2], Stacey B. Andersen[2], Xin Mei[2,3], Quan H. Nguyen[2†] and Joseph E. Powell[1,4,5*†]

[1]Garvan Institute of Medical Research, Sydney, Australia and [2]Institute of Molecular Bioscience, University of Queensland, Brisbane, Australia and [3]South China Botanical Garden, Chinese Academy of Sciences, Guangzhou, China and [4]School of Medical Sciences, University of New South Wales, Sydney, Australia and [5]Garvan-Weizmann Centre for Cellular Genomics, Garvan Institute of Medical Research, Sydney, Australia

[*]Corresponding author: j.powell@garvan.org.au
[†]Joint senior authors

Abstract

Background Recent developments in single cell RNA sequencing (scRNA-seq) platforms have vastly increased the number of cells typically assayed in an experiment. Analysis of scRNA-seq data is multidisciplinary in nature, requiring careful consideration of the application of statistical methods with respect to the underlying biology. Few analysis packages exist that are at once robust, computationally fast, and allow flexible integration with other bioinformatics tools and methods. **Findings** *ascend* is an R package comprised of tools designed to simplify and streamline the preliminary analysis of scRNA-seq data, while addressing the statistical challenges of scRNA-seq analysis, while enabling flexible integration with genomics packages and native R functions, including fast parallel computation and efficient memory management. The package incorporates both novel and established methods to provide a framework to perform cell and gene filtering, quality control, normalization, dimension reduction, clustering, differential expression, and a wide-range of visualization functions. **Conclusions** *ascend* is designed to work with scRNA-seq data generated by any high-throughput platform, and includes functions to convert data objects between software packages. The *ascend* workflow is simple and interactive, suitable for implementation by a broad range of users, including those with little programming experience.

Key words: single cell; scRNA-seq; filtering; clustering; normalization; differential expression; data visualization; R package

Findings

Background

Single cell RNA sequencing (scRNA-seq) has revolutionized the way we understand the transcriptional programs of cells. Recent advances in barcoding molecular biology techniques, coupled with microfluidics have yielded platforms such as 10x Genomics Chromium [1] and Drop-seq [2], which are capable of capturing the transcriptomes of tens of thousands of single cells simultaneously. The increased capacity of scRNA-seq has been advantageous as larger sample sizes provide greater statistical power, and correspondingly higher resolution to determine differences in cellular features. A consequence has been the increase in the complexity of scRNA-seq data, creating new challenges for data management, statistical methods, data visualization, and computing strategies. A number of scRNA-seq specific methods and toolkits have been developed to address

Compiled on: June 26, 2019.
Draft manuscript prepared by the author.

Key Points

- *ascend* is a fast and easy-to-use software for thorough and interactive analysis of scRNA-seq data.
- *ascend*'s streamlined workflow includes filtering, normalization, dimension reduction, clustering, differential expression and visualization.
- *ascend* optimizes parallelization and algorithms for improving speed of each analysis step. e.g. differential expression analysis
- *ascend* implements Clustering by Optimal REsolution (CORE) for unsupervised, robust hierarchical clustering.

these challenges ([3], [4] and [5]), but both the functionality, and specific methods implemented vary. It is becoming apparent that for a given scRNA-seq dataset, the specific analysis steps need to be carefully considered in light of the underlying biology. For single cell analysis packages, flexibility in both the choice of methods implemented and arguments passed to functions is therefore important.

Here we present *ascend*, an R package designed to create a simple and streamlined workflow for the analysis of scRNA-seq experiments. *ascend* is designed to handle data generated from any single cell library preparation platform; this can include data from single and paired-end reads, and optionally, with unique molecular identifiers (UMIs). *ascend* imports scRNA-seq data following the generation of an expression matrix consisting of transcript counts from each cell, and performs user-friendly quality control, filtering, normalization, dimension reduction, clustering, differential expression and visualization. It includes functions to leverage multiple CPUs, allowing most analyses to be performed on a standard desktop or laptop.

Data object

The foundation of the *ascend* R package is the **E**xpression and **M**etadata **Set**, a data container class that inherits from the SingleCellExperiment superclass [6]. The SingleCellExperiment class, from the Bioconductor R package of the same name, was introduced as a container class specifically for single cell genomics data. It is structured in the context of the gene-cell expression matrix and contains slots that can hold data that may be used in scRNA-seq analysis - specifically spike-in information, normalization factors, transformations of the original count data, metadata and data related to cells and genes.

The EMSet deviates from the SingleCellExperiment in which it is a dynamic element. The object is always accompanied by a set of quality control metrics that is reflective of the data that is currently stored in the counts slot of the object. These values are automatically recalculated by the package whenever changes are made to the count matrix, which occurs during batch normalisation and filtering. Another feature of the EMSet is the logging of operations, ensuring analysis is performed in the correct order and allowing users to review changes. As metadata can play a key role in functions such as plotting and differential expression analysis, we have separated cell-related and gene-related metadata from calculated values by storing them in dedicated slots introduced by the EMSet. Additional slots have also been introduced to store objects related to clustering and differential expression analysis.

The EMSet retains the convenient row and column subsetting operations of the SingleCellExperiment and introduces methods to manipulate the object based on conditions defined in the cell metadata slot. To ensure compatibility with other software packages that also use the SingleCellExperiment class, a conversion function is supplied to preserve data stored in EMSet-specific slots. This data can then be retrieved when converting back to an EMSet.

Batch normalization

Typically, samples comprising of libraries of thousands of cells are often processed in separate batches and the resulting data require aggregation before analysis. This can introduce systematic biases due to technical variation in each batch. To address this, *ascend* provides simple and fast methods to normalize between batches, (*normaliseBatches*). To perform fast batch-to-batch normalisation, we calculate a scaling factor for each batch and multiply the expression values to a batch-specific constant. The batch scale factor is the ratio of the median sequencing reads among all batches to the total reads of a batch. This scaling approach overcomes the limitation of reducing read depths in all libraries to the lowest-depth library, which is commonly applied in the global scaling method. We also introduce more cell-to-cell normalisation options in the later section.

Filtering and quality control

Quality control (QC) is an important step of scRNA-seq data analysis, as it can be used to reduce poor quality data that may mask biologically significant variation [7]. Sources of technical noise include low quality cells that are generally defined as empty droplets, droplets with multiple cells, and dead or dying cells [8]. The quality of cells is described by a series of metrics, such as total number of reads, number of genes expressed by a cell, mean gene expression of a gene or a cell and proportion of a gene's expression to total expression. Low quality cells are identified as outliers in terms of library size and gene expression or with expression dominated by controls that are usually defined as mitochondrial and ribosomal genes. As the EMSet automatically recalculates QC metrics when changes are made to the count matrix, the quality of the dataset can be monitored in real-time with the aid of quality control plots (*plotGeneralQC*). Users can also review the EMSet log for a record of cells and genes removed by filtering methods. Since these QC steps should allow a user to filter cells or genes based on their own defined metrics, *ascend*'s QC functions allow arguments to be passed using additional metadata.

Cell-cell normalization

Cell-cell normalization is another crucial step to remove technical variation between individual cells. The normalization of scRNA-seq data is complicated by the zero-inflated count distributions of genes, that may be due to either biological or technical factors. *ascend* addresses this issue by adapting the normalization by Relative Log Expression (RLE) method[9] for zero-inflated data by estimating size factors from the geometric means based on true count values that are greater than zero. For each gene, a gene-specific geometric mean is estimated

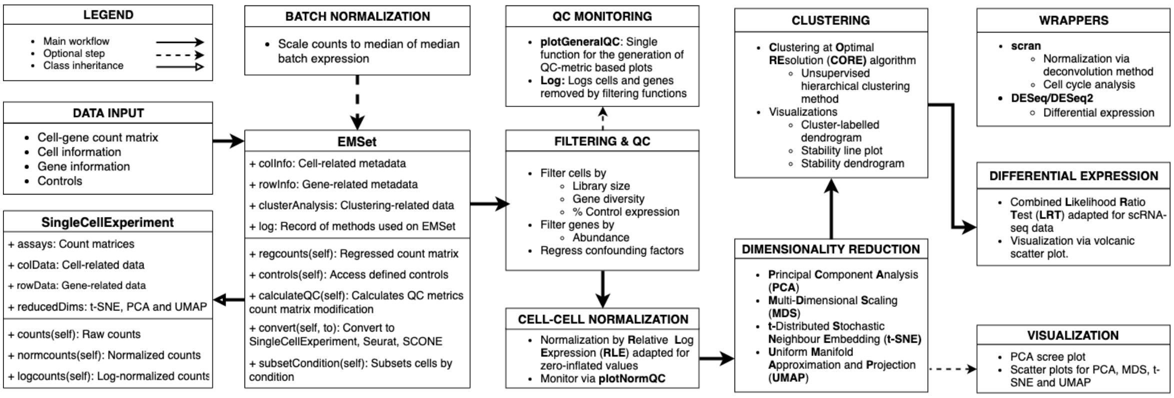

Figure 1. A summary of the typical analysis workflows and major function groups available in *ascend*.

across all cells, not including cells with zero values. The cell-specific size factor is then calculated based on the expression of the gene in a cell relative to the geometric mean of that gene. The size factors for all genes in a cell are used for calculating the cell-specific size factor. We introduce the use of scran wrapper function as the default normalisation method, and recommend this method if the computation time and memory are not limiting factors. Alternatively, the RLE approach introduced here is the faster and more memory efficient option for cell-to-cell normalisation. Users can review the impact of normalization on the counts by generating a series of plots with the *plotNormQC* function, that compares pre-normalized and normalized library sizes and individual gene counts.

Reduction of high-dimensional space

Since scRNA-seq data is typically multiple orders of magnitude larger than bulk RNA-seq data (n-cells x m-genes), dimensionality reduction is vital. Moreover, the expression levels of many genes are likely to be correlated, therefore the problem of collinearity is common, while additional factors such as dropout rate, and high expression variation increase noise in the data [10]. *ascend* contains functions to perform principal component analysis (PCA) to reduce the dimensions of the normalized count data and preserve the data structure (i.e. explain the majority of the variance between cells). t-SNE (t-distributed Stochastic Neighbor Embedding), and Multi-Dimensional Scaling (MDS) are only used to visualize cells in a low-dimensional space, supplemented by information supplied by the user or generated by downstream analysis.

Clustering

Clustering cells into subpopulations or subtypes provides structure to the dataset by grouping transcriptionally similar cells. *ascend* implements our previously published CORE method [11], which identifies the most stable clustering identity. Firstly, a Euclidean distance matrix between cells is calculated from the first 20 principal components of the PCA-reduced normalized count matrix. An unsupervised dendrogram is then constructed by applying hierarchical clustering. Outlier cells identified by this initial round of clustering removed from the dataset, although their identifiers are retained in the EMSet logs. The dendrogram is then dynamically re-clustered by a top-down split and merging process over multiple iterations with changing tree-height thresholds. This approach merges smaller clusters into larger consensus clusters, and uses an adjusted Rand index to compare different clustering results to identify the most stable number of clusters. The method is fast and scalable, enabling the analysis of small clusters at high resolution, or larger clusters for more general classification with simpler downstream analysis.

Differential expression

In a heterogeneous dataset, such as scRNA-seq data, analyzing the differentially expressed (DE) genes between one cluster and the combined remaining clusters can reveal signature genes that can be used to assign identity to a population of cells, or to more clearly understand cell transition states. After decomposing the data into subpopulations, *ascend* provides a combined Likelihood Ratio Test (LRT) to compare these subpopulations by finding biological signatures that distinguish them, taking into account subpopulation-size imbalance and high drop-out rates. Introduced as a method for single-cell qPCR data[12], the combined LRT has been adapted in *ascend* such that it takes into account genes with zero variance. LRT is particularly suitable for the cases where the number of cells in two clusters are very different. In these cases, most dispersion estimation methods, such as those in DESeq do not result in a convergence. The imbalance issue becomes exaggerated for the cases of smaller clusters, where the high dropout rates have a higher impact. LRT uses a combined distribution assumption consisting of both discrete (on/off) and continuous (low/high expression) components, which helps overcome the issues in dropout and small number of cells. LRT applies Chi-Square approximation for likelihood differences and thus is fast and less memory-intensive. A similar LRT test approach that optimizes a two-part general linearized model to estimate parameters that account for bimodality and stochastic dropout (cell detection rate) implemented in the MAST package is more computationally intensive, especially for datasets with large cell numbers [13]. The LRT applied in ascend does not model cell detection rate to use as a covariate when comparing subpopulations. The resulting implementation is fast, scalable and robust, even in situations where standard DE methods fail. Wrapper functions are also provided for DE analysis based on negative binomial tests from DESeq (Anders and Huber, 2010). We introduced several modifications that allow (i) more accurate estimation of fold change (adjusted fold change), and (ii) more efficient multiprocessing, using a divide and conquer approach, to handle large datasets and substantially reduce computational time.

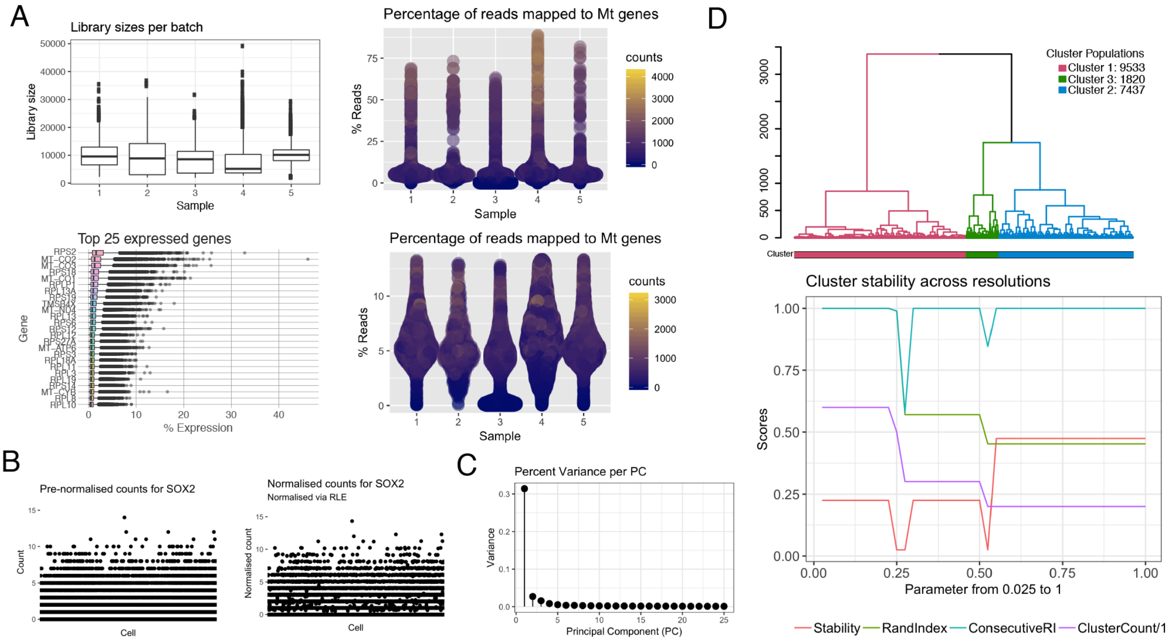

Figure 2. Graphics generated by *ascend* during different stages of analysis. A: Quality control plots include a boxplot representing distribution of library sizes across each batch, a boxplot representing the expression of the top 25 most abundant transcripts and violin plots representing proportion of mitochondrial-related transcripts to total expression per sample. B: Normalization quality control plot represents the expression of the SOX2 gene before and after RLE normalization. C: Scree plot related to Principal Component dimensionality reduction. D: Clustering plots include a cluster-labeled dendrogram and a line plot depicting the relationships between cluster numbers and stability.

Benchmarking

The CPU time of the ascend package was compared to two other toolkits developed for scRNA-seq analysis – *Seurat*[14] and *scater*[4]. Using a dataset that comprised of 1,272 retinal ganglion cells from the study by Daniszewski et al. [15], these packages were used to perform quality control, normalization, dimensionality reduction, clustering and differential expression using equivalent methods. As shown in Supplementary File 1, ascend's processing time is comparable to *Seurat*[14] and *scater*[4].

Conclusion

In summary, *ascend* is a user-friendly and computationally efficient package for analyzing scRNA-seq data from all experimental platforms. *ascend* implements quality control and filtering approaches that are highly customizable, a unsupervised clustering method (CORE), and optimizes speed for implementing established analysis techniques for normalization and differential gene expression. Statistical methods for single-cell analysis are constantly evolving. Here we have implemented a series of current cutting-edge approaches, although the flexibility of *ascend* allows it to adapt as future methods are developed. The *ascend* package and context-specific tutorials addressing a range of analytical scenarios are available at https://github.com/powellgenomicslab/ascend. We expect that *ascend* is especially useful for biologists who wish to explore their own datasets using expert domain knowledge and an easy-to use and complete toolkit.

Methods

Data

Here we present an application case study of *ascend* using scRNA-seq data from undifferentiated human induced Pluripotent Stem Cells (hiPSCs) generated as described by Nguyen and Lukowski *et al.* [11]. The raw 10x Chromium Single Cell 3' Gene Expression dataset consists of 20,448 cells that are divided into five samples. Raw (FASTQ or aggregated count matrix) and processed data can be downloaded from ArrayExpress (accession number: E-MTAB-6687).

Preprocessing of scRNA-seq dataset

The raw expression data from each sample were combined into a single dataset using Chromium's Cell Ranger 1.2.0 *aggr* function. This function performs two tasks – batch normalization and transcript count aggregation. Cell Ranger first normalizes the sequencing depth between the five samples by subsampling reads for each sample until their median depth equals the sample with the shallowest read depth. Once normalized, the transcript counts from each sample are combined into a single matrix. The rows of this matrix were labeled with ENSEMBL gene identifiers; to simplify analysis, these were replaced with corresponding gene names that were stored in the Cell Ranger outputs.

"ascend" analysis

ascend was run in RStudio (R Version 3.5.0), and analysis of data from quality control to differential expression (LRT) took 95 minutes on a MacBook Pro laptop with a dual-core Intel Core i5 2.7GHz and 8GB of RAM. To minimize memory use, the raw expression matrix was converted into a sparse matrix using the Matrix R Package [16]. The sparse matrix and accompanying cell-related metadata were loaded into an *EMSet*.

```
library(ascend)
```

```
library(Matrix)

# Read in file downloaded from ArrayExpress
counts <- read.csv("raw_expMat_cellbarcodes.tsv",
sep = "\t")

# Convert counts to a sparseMatrix
counts <- as(as.matrix(counts), "dgCMatrix")

# Create colInfo dataframe
batches <- sapply(strsplit(colnames(counts),
"[.]"), function(x) x[2])

colInfo <- S4Vectors::DataFrame(
cell_barcode = colnames(counts),
batch = batches)

# Define controls
mt_genes <- grep("^Mt-",
rownames(counts),
ignore.case = TRUE,
value = TRUE)

rb_genes <- grep("^Rps|^Rpl",
rownames(counts),
ignore.case = TRUE,
value = TRUE)

controls <- list(Mt = mt_genes,
                 Rb = rb_genes)

# Create a new EMSet
EMSet <- EMSet(counts,
colInfo = colInfo,
controls = controls)
```

The quality of the expression data was assessed with the aid of quality control figures generated by the *plotGeneralQC* function from *ascend* package.

```
raw_qc_plots <- plotGeneralQC(EMSet)
```

The data then underwent quality control. First, cells were filtered based on library size, number of detected genes and reads mapped to mitochondrial and ribosomal genes using the default threshold of 3 x MAD range. Next, cells were removed if 20% of reads were mapped to mitochondrial transcripts and 50% of reads were mapped to ribosomal transcripts. Finally, genes were removed if they were expressed in less than 0.1% of the cell population.

```
# Remove cells that are outliers
EMSet <- filterByOutliers(EMSet,
cell.threshold = 3,
control.threshold = 3)

# Remove cells where mitochondrial-related
# transcripts account for at least 20% of reads
EMSet <- filterByControl(EMSet,
control = "Mt",
pct.threshold = 20)

# Remove cells where ribosomal-related
# transcripts account for at least 50% of reads
EMSet <- filterByControl(EMSet,
control = "Rb",
pct.threshold = 50)
# Remove genes that are expressed in less than
# 0.1% of the cell population
EMSet <- filterLowAbundanceGenes(EMSet, pct.threshold = 0.1)
```

QC removed 1,683 cells and 16,272 genes, leaving 18,765 cells and 16,466 genes for further analysis. The UMI counts for the remaining cells and genes were normalized with the *normaliseByRLE* function. The effectiveness of the normalization method was assessed with the aid of figures generated by the *plotNormQC* function. Mitochondrial and ribosomal gene transcripts were removed from the data before proceeding with further analysis.

```
# Normalize dataset using RLE
EMSet <- normaliseByRLE(EMSet)

# Plot normalisation quality control plots
norm_qc <- plotNormQC(EMSet,
gene_list = c("GAPDH", "MALAT1"))

# Remove controls from dataset
EMSet <- excludeControl(EMSet, control = c("Mt", "Rb"))
```

To reduce the dimensions of the data, the normalized UMI count matrix was reduced using the *ascend* function *runPCA*. This function is a wrapper for *prcomp* function from the irlba R package.

```
# Reduce dataset with PCA
EMSet <- runPCA(EMSet, ngenes = 1500, scaling = TRUE)
```

The scree plot generated by *ascend*'s *plotPCAVariance* function revealed the first 10 principal components (PCs) explained 88.70% of the variance in this data. These 10 PCs were passed to the CORE algorithm function to build a cell distance matrix, and subsequently a dendrogram that was used to identify clusters.

```
EMSet <- runCORE(EMSet,
conservative = FALSE,
remove.outliers = TRUE,
nres = 40,
dims = 10)
```

Using the default arguments, the CORE method generated clustering results for 40 different resolutions, and based on the Rand index, the function identified three clusters of cells that represent the most stable result. Clusters 1, 2 and 3 comprised of 9046, 6436 and 3283 cells respectively.

To characterize the biological properties of the three clusters, differential expression was performed using *ascend*'s *runDiffExpression* function. The expression of each cluster was compared to the expression of the other clusters.

```
# Comparison of cluster 1 vs other clusters
cluster1_vs_all <- runDiffExpression(EMSet,
group = "cluster",
    condition.a = 1,
    condition.b = c(2, 3))

# Comparison of cluster 2 vs other clusters
cluster2_vs_all <- runDiffExpression(EMSet,
group = "cluster",
    condition.a = 2,
    condition.b = c(1, 3))

# Comparison of cluster 3 vs other clusters
cluster3_vs_all <- runDiffExpression(EMSet,
group = "cluster",
    condition.a = 3,
    condition.b = c(1, 2))
```

Using a Bonferroni-corrected P-value threshold ($P < 3.1 \times 10^{-7}$) and an absolute $\log_2$ fold change greater than 2, differential expression analysis revealed clusters 1, 2, and 3 respectively had 230, 7, and 47 differentially expressed genes.

Availability of source code and requirements

- Project name: *ascend*
- Project home page: https://github.com/powellgenomicslab/ascend
- Operating system(s): Platform independent
- Programming language: R
- Other requirements: R 3.5, Bioconductor 3.7
- License: GPL 3.0
- RRID: SCR_017257

Availability of supporting data and materials

The data supporting the results of this article are available in ArrayExpress at https://www.ebi.ac.uk/arrayexpress/experiments/E-MTAB-6687/[17].

An archival copy of the code and supporting data is also available via the GigaScience repository, GigaDB10.5524/100615 [18]

Declarations

None declared.

List of abbreviations

scRNA-seq: single cell RNA-sequencing; RNA-seq: RNA-sequencing; CORE: clustering at optimal resolution; PCA: principal component analysis; t-SNE: t-distributed Stochastic Neighbor Embedding; MDS: multi-dimensional scaling; RLE: relative log expression; UMI: unique molecular identifier.

Ethical Approval

Not applicable.

Consent for publication

Not applicable.

Competing Interests

The author(s) declare that they have no competing interests.

Funding

This work was supported by the National Health and Medical Research Council grants 1107599 and 1083405.

Author's Contributions

AS wrote the software; all authors contributed to software development; AS, SWL, QHN and JEP wrote the manuscript. QHN and JEP oversaw the project.

References

1. Zheng GXY, Terry JM, Belgrader P, Ryvkin P, Bent ZW, Ziraldo SB, et al. Massively parallel digital transcriptional profiling of single cells. Nature Communications 2017;8(206):667–3170.
2. Macosko EZ, Basu A, Satija R, Nemesh J, Shekhar K, Goldman M, et al. Highly parallel genome-wide expression profiling of individual cells using nanoliter droplets. Cell 2015;161(5):1202–1214.
3. Butler A, Hoffman P, Smibert P, Papalexi E, Satija R. Integrating single-cell transcriptomic data across different conditions, technologies, and species analysis. Nature Biotechnology 2018;36(5).
4. McCarthy DJ, Campbell KR, Lun ATL, Wills QF. Scater: Pre-processing, quality control, normalization and visualization of single-cell RNA-seq data in R. Bioinformatics 2017;33(8):1179–1186.
5. Lun ATL, Bach K, Marioni JC. Pooling across cells to normalize single-cell RNA sequencing data with many zero counts. Genome Biology 2016;17.
6. Lun A, Risso D. SingleCellExperiment: S4 Classes for Single Cell Data; 2018, r package version 1.3.6.
7. Bacher R, Kendziorski C, Auer P, Doerge R, Robles J, Qureshi S, et al. Design and computational analysis of single-cell RNA-sequencing experiments. Genome Biology 2016;17(1):63.
8. Ilicic T, Kim JK, Kolodziejczyk AA, Bagger FO, McCarthy DJ, Marioni JC, et al. Classification of low quality cells from single-cell RNA-seq data. Genome Biology 2016;17(1).
9. Anders S, Huber W. Differential expression analysis for sequence count data. Genome Biology 2010;11(10):R106.
10. Hicks SC, Townes FW, Teng M, Irizarry RA. Missing data and technical variability in single-cell RNA-sequencing experiments. Biostatistics 2017 Nov;.
11. Nguyen Q, Lukowski S, Chiu H, Senabouth A, Bruxner T, Christ A, et al. Single-cell RNA-seq of human induced pluripotent stem cells reveals cellular heterogeneity and cell state transitions between subpopulations. Genome research 2018 jul;28(7):gr.223925.117.
12. McDavid A, Finak G, Chattopadyay PK, Dominguez M, Lamoreaux L, Ma SS, et al. Data exploration, quality control and testing in single-cell qPCR-based gene expression experiments. Bioinformatics 2013 feb;29(4):461–467.
13. Finak G, McDavid A, Yajima M, Deng J, Gersuk V, Shalek AK, et al. MAST: a flexible statistical framework for assessing transcriptional changes and characterizing heterogeneity in single-cell RNA sequencing data. Genome Biology 2015;16:278. https://www.ncbi.nlm.nih.gov/pmc/articles/PMC4676162/.
14. Satija R, Farrell JA, Gennert D, Schier AF, Regev A. Spatial reconstruction of single-cell gene expression data. Nature Biotechnology 2015;33(5):495–502. https://www.nature.com/articles/nbt.3192.pdf.
15. Daniszewski M, Senabouth A, Nguyen Q, Crombie DE, Lukowski SW, Kulkarni T, et al. Single Cell RNA Sequencing of stem cell-derived retinal ganglion cells. bioRxiv 2017;http://biorxiv.org/content/early/2017/09/22/191395.
16. Bates D, Maechler M. Matrix: Sparse and Dense Matrix Classes and Methods; 2018, https://CRAN.R-project.org/package=Matrix, r package version 1.2-14.
17. ;https://www.ebi.ac.uk/arrayexpress/experiments/E-MTAB6687/.
18. 5525/100615, Senabouth A, Lukowski S, Hernandez J, Andersen S, Mei X, et al. ascend: R package for analysis of single cell RNA-seq data. GigaScience Database 2019;http://dx.doi.org/10.5524/100615.
