# Supplementary material for: ascend: R package for analysis of single-cell RNA-seq data
Source: Gigascience. 2019 Aug 24;8(8):giz087. doi: 10.1093/gigascience/giz087 (PMC6735844; doi:10.1093/gigascience/giz087)
Supplement: giz087_Response_to_Reviewer_Comments_Original_Submission [file giz087_response_to_reviewer_comments_original_submission.pdf]

Author's Response To Reviewer Comments

Close

We would like to thank the referees again for their time reviewing our manuscript and providing additional helpful comments. We have addressed all comments and included a point-by-point reply below.

Reviewer reports:
Reviewer #1: The authors have addressed my concerns.

Reviewer #2: It is good to have alternative workflows for single-cell analysis, and I am glad to see the authors have submitted the package to Bioconductor. I hope the authors maintain the package and update with new methods as necessary such as if new normalizations or batch corrections are developed. I only have two comments that I hope the authors try to clarify further.

1. The statement starting with "Optionally, after batch-to-batch normalisation, we also…" should not be in that location. It seems to suggest to readers that this is the recommended method, whereas later that is not the case. In these sentences the manuscript also claims that this normalization approach is more "robust" without providing any evidence or citation.

Thank you for highlighting this. We have amended this section, removing mention of the batch-batch normalisation method here.

2. It's still not completely clear to me how the authors extension of the sc-qPCR method is different from MAST. The same authors of the qPCR method extended it here: "MAST: a flexible statistical framework for assessing transcriptional changes and characterizing heterogeneity in single-cell RNA sequencing data". MAST is also an LRT, but I am assuming that here you are not using the detection rate as a covariate? That's OK if true, it just needs to be clear to the reader. I imagine this could be a frequently asked question by users down the road, so even a sentence on how it is different from (or similar to) MAST would help.

We have amended this section to include a couple of sentences on the relationship of the LRT we use to that in MAST. LRT applies Chi-Square approximation for likelihood differences and thus is fast and less memory-intensive. A similar LRT test approach that optimizes a two-part general linearized model to estimate parameters that account for bimodality and stochastic dropout (cell detection rate) implemented in the MAST package is more computationally intensive, especially for datasets with large cell numbers. The LRT applied in ascend does not model cell detection rate to use as a covariate when comparing subpopulations.

3. Suggestion only: I may have missed it, but it might be helpful to include a statement that says something like "Statistical methods for single-cell analysis are constantly evolving. Here we have implemented XX. The flexibility of ascend allows it to adapt as future methods are developed and prove useful".

Thank you for this suggestion, we have included the following sentence in the conclusion stating "Statistical methods for single-cell analysis are constantly evolving. Here we have implemented a series of current cutting-edge approaches, although the flexibility of ascend allows it to adapt as future methods are developed"

Close
