# Supplementary material for: ascend: R package for analysis of single-cell RNA-seq data
Source: Gigascience. 2019 Aug 24;8(8):giz087. doi: 10.1093/gigascience/giz087 (PMC6735844; doi:10.1093/gigascience/giz087)
Supplement: giz087_Response_to_Reviewer_Comments_Revision_1 [file giz087_response_to_reviewer_comments_revision_1.pdf]

Author's Response To Reviewer Comments

Close

Editor requested edits made

Close
