# Supplementary material for: ascend: R package for analysis of single-cell RNA-seq data
Source: Gigascience. 2019 Aug 24;8(8):giz087. doi: 10.1093/gigascience/giz087 (PMC6735844; doi:10.1093/gigascience/giz087)
Supplement: giz087_Response_to_Reviewer_Comments_Revision_2 [file giz087_response_to_reviewer_comments_revision_2.pdf]

# Author's Response To Reviewer Comments

Close

Dear Hans,

the following action items have been completed.

1. the following has been added to the data availability section "An archival copy of the code and supporting data is also available via the GigaScience repository, GigaDB10.5524/100615"
2. A citation to the GigaDB doi to the bibliography has been added.
3. The ArrayExpress URL has been added to the bibliography

Many thanks,

// Joseph

Close
