# Supplementary material for: ascend: R package for analysis of single-cell RNA-seq data
Source: Gigascience. 2019 Aug 24;8(8):giz087. doi: 10.1093/gigascience/giz087 (PMC6735844; doi:10.1093/gigascience/giz087)
Supplement: giz087_Reviewer_2_Report_Original_Submission — Rhonda Bacher -- 5/14/2019 Reviewed [file giz087_reviewer_2_report_original_submission.pdf]

Reviewer Report

Title: ascend: R package for analysis of single cell RNA-seq data

Version: Original Submission **Date:** 5/14/2019

Reviewer name: Rhonda Bacher

Reviewer Comments to Author:

It is good to have alternative workflows for single-cell analysis, and I am glad to see the authors have submitted the package to Bioconductor. I hope the authors maintain the package and update with new methods as necessary such as if new normalizations or batch corrections are developed. I only have two comments that I hope the authors try to clarify further:

1. The statement starting with "Optionally, after batch-to-batch normalisation, we also…" should not be in that location. It seems to suggest to readers that this is the recommended method, whereas later that is not the case. In these sentences the manuscript also claims that this normalization approach is more "robust" without providing any evidence or citation.

2. It's still not completely clear to me how the authors extension of the sc-qPCR method is different from MAST. The same authors of the qPCR method extended it here: "MAST: a flexible statistical framework for assessing transcriptional changes and characterizing heterogeneity in single-cell RNA sequencing data". MAST is also an LRT, but I am assuming that here you are not using the detection rate as a covariate? That's OK if true, it just needs to be clear to the reader. I imagine this could be a frequently asked question by users down the road, so even a sentence on how it is different from (or similar to) MAST would help.

Suggestion only: I may have missed it, but it might be helpful to include a statement that says something like "Statistical methods for single-cell analysis are constantly evolving. Here we have implemented XX. The flexibility of ascend allows it to adapt as future methods are developed and prove useful".

Level of Interest

Please indicate how interesting you found the manuscript: Choose an item.

Quality of Written English

Please indicate the quality of language in the manuscript: Choose an item.

Declaration of Competing Interests

Please complete a declaration of competing interests, considering the following questions:

- Have you in the past five years received reimbursements, fees, funding, or salary from an organisation that may in any way gain or lose financially from the publication of this manuscript, either now or in the future?

- Do you hold any stocks or shares in an organisation that may in any way gain or lose financially from the publication of this manuscript, either now or in the future?
- Do you hold or are you currently applying for any patents relating to the content of the manuscript?
- Have you received reimbursements, fees, funding, or salary from an organization that holds or has applied for patents relating to the content of the manuscript?
- Do you have any other financial competing interests?
- Do you have any non-financial competing interests in relation to this paper?

If you can answer no to all of the above, write 'I declare that I have no competing interests' below. If your reply is yes to any, please give details below.

I declare that I have no competing interests.

I agree to the open peer review policy of the journal. I understand that my name will be included on my report to the authors and, if the manuscript is accepted for publication, my named report including any attachments I upload will be posted on the website along with the authors' responses. I agree for my report to be made available under an Open Access Creative Commons CC-BY license (http://creativecommons.org/licenses/by/4.0/). I understand that any comments which I do not wish to be included in my named report can be included as confidential comments to the editors, which will not be published.

I agree to the open peer review policy of the journal

To further support our reviewers, we have joined with Publons, where you can gain additional credit to further highlight your hard work (see: https://publons.com/journal/530/gigascience). On publication of this paper, your review will be automatically added to Publons, you can then choose whether or not to claim your Publons credit. I understand this statement.

Yes Choose an item.
